# Prediction for early recurrence of intrahepatic mass-forming cholangiocarcinoma: quantitative magnetic resonance imaging combined with prognostic immunohistochemical markers

**DOI:** 10.1186/s40644-019-0234-4

**Published:** 2019-07-15

**Authors:** Li Zhao, Xiaohong Ma, Meng Liang, Dengfeng Li, Peiqing Ma, Sicong Wang, Zhiyuan Wu, Xinming Zhao

**Affiliations:** 1 Department of Diagnostic Radiology, National Cancer Center/Cancer Hospital, Chinese Academy of Medical Sciences and PekingUnion Medical College, No.17, Panjiayuan Nanli, Chaoyang District, Beijing, 100021 China; 2 Department of Pathology, National Cancer Center/Cancer Hospital, Chinese Academy of Medical Sciences and PekingUnion Medical College, No.17, Panjiayuan Nanli, Chaoyang District, Beijing, 100021 China; 3 Department of Pharmaceutical Diagnosis, GE Healthcare, Life Sciences, No.1 Tongji South Road, Beijing, 100176 China; 4 State Key Laboratory of Molecular Oncology, National Cancer Center/Cancer Hospital, Chinese Academy of Medical Sciences and PekingUnion Medical College , No.17, Panjiayuan Nanli, Chaoyang District, Beijing, 100021 China

**Keywords:** Liver neoplasms, Cholangiocarcinoma, MRI, Immunohistochemistry, Recurrence, Radiomics

## Abstract

**Background:**

Partial hepatectomy is the first option for intrahepatic mass-forming cholangiocarcinoma (IMCC) treatment, which would prolong survival. The main reason for the poor outcome after curative resection is the high incidence of early recurrence (ER). The aim of this study was to investigate the combined predictive performance of qualitative and quantitative magnetic resonance imaging (MRI) features and prognostic immunohistochemical markers for the ER of IMCC.

**Methods:**

Forty-seven patients with pathologically proven IMCC were enrolled in this retrospective study. Preoperative contrast-enhanced MRI and post-operative immunohistochemical staining of epidermal growth factor receptors (EGFR), vascular endothelial growth factor receptor (VEGFR), P53 and Ki67 were performed. Univariate analysis identified clinic-radiologic and pathological risk factors of ER. Radiomics analysis was performed based on four MRI sequences including fat suppression T2-weighted imaging (T2WI/FS), arterial phase (AP), portal venous phase (PVP), and delayed phase (DP) contrast enhanced imaging. A clinicoradiologic-pathological (CRP) model, radiomics model, and combined model were developed. And ROC curves were used to explore their predictive performance for ER stratification.

**Results:**

Enhancement patterns and VEGFR showed significant differences between the ER group and non-ER group (*P* = 0.001 and 0.034, respectively). The radiomics model based on AP, PVP and DP images presented superior AUC (0.889, 95% confidence interval (CI): 0.783–0.996) among seven radiomics models with a sensitivity of 0.938 and specificity of 0.839. The combined model, containing enhancement patterns, VEGFR and radiomics features, showed a preferable ER predictive performance compared to the radiomics model or CRP model alone, with AUC, sensitivity and specificity of 0.949, 0.875 and 0.774, respectively.

**Conclusions:**

The combined model was the superior predictive model of ER. Combining qualitative and quantitative MRI features and VEGFR enables ER prediction, thus facilitating personalized treatment for patients with IMCC.

## Background

Intrahepatic cholangiocarcinoma (ICC) is the second most common primary liver malignancy and is arising in incidence worldwide [1, 2]. It originates from the intrahepatic biliary epithelium and can be classified into three types according to the morphologic classification system: mass forming, periductal infiltrating, and intraductal growing [3]. Intrahepatic mass-forming cholangiocarcinoma (IMCC) accounts for a large percentage of ICC. Partial hepatectomy is the first option for IMCC curative treatment, which would prolong survival [4]. However, even after a curative resection, the 5-year survival rate is only 20–35% [5, 6]. The main reason for the poor outcome is the incidence of recurrence, which can be as high as 54–71% [5, 7, 8].

The time interval from the resection to IMCC recurrence is an independent prognostic factor of survival [9]. Approximately 78.8% of recurrence develops within 24 months, defined as early recurrence (ER), and the prognosis for patients with ER is worse than that for those with late recurrence. Adjuvant trans-arterial chemoembolization (TACE) or chemotherapy after surgery was associated with better survival among the IMCC patients with early recurrence [10, 11]. Therefore, patients at a high risk of ER need to be precisely determined and effective adjunctive treatment strategies and closer follow-up after operation need to be performed.

Previous studies have revealed several pathological tumor characteristics associated with postoperative ER of IMCC (e.g. tumor size, satellite lesions, lymph node metastasis, lymphatic invasion, microvascular invasion and stage) [9]. In addition, certain immunohistochemical molecules have been reported as predictive markers of ICC prognosis. Iguchi et al. found that P53 and Ki67, markers indicating cell proliferation, were related to the overall survival of ICC patients [12]. Epidermal growth factor receptor (EGFR) expression was demonstrated to be an independent predictor of ICC prognosis [13]. As an anti-angiogenesis therapeutic target, vascular endothelial growth factor receptor (VEGFR) expression has been correlated with the prognosis of many cancers (e.g. breast cancer, ovarian cancer, lung cancer, lymphoma, etc.) [14–17]. However, ER prediction by these factors leads to noticeably different outcomes among studies. Therefore, it is necessary to further explore whether they are effective predictors of ER in IMCC.

Magnetic resonance imaging (MRI) is widely used in the diagnosis and treatment planning of liver tumors. Previous studies revealed some radiological features that might be predictors of IMCC prognosis, such as the degree of diffusion restriction on diffusion weighted images (DWI), enhancement pattern of contrast enhanced MRI (CE-MRI), and intensity on the hepatobiliary phase of gadoxetic acid-enhanced MRI [18–20]. However, these qualitative assessments were unable to quantify tumor heterogeneity. Radiomics is considered to be an emerging quantitative technique for evaluating the entire underlying intra-tumor heterogeneity by extracting numerous features from radiologic images. Studies in many cancers (e.g. colorectal cancer, breast cancer, lung cancer, esophageal cancer and hepatocellular carcinoma) have shown that radiomics has the potential for prognosis prediction [21–25].

We combined the above pathological characteristics and immunohistochemical molecules with both visible and invisible radiological features for better ER prediction. To our knowledge, combining immunohistochemistry and radiomics features for ER prediction in IMCC patients has not yet been investigated. Therefore, the aim of this study was to develop a nomogram based on radiological features, immunohistochemical markers, and radiomics features for predicting the ER of IMCC to create a better stratification of IMCC patients thus improving personalized treatment.

### Materials and methods

### Patients

**Fig. 1:**
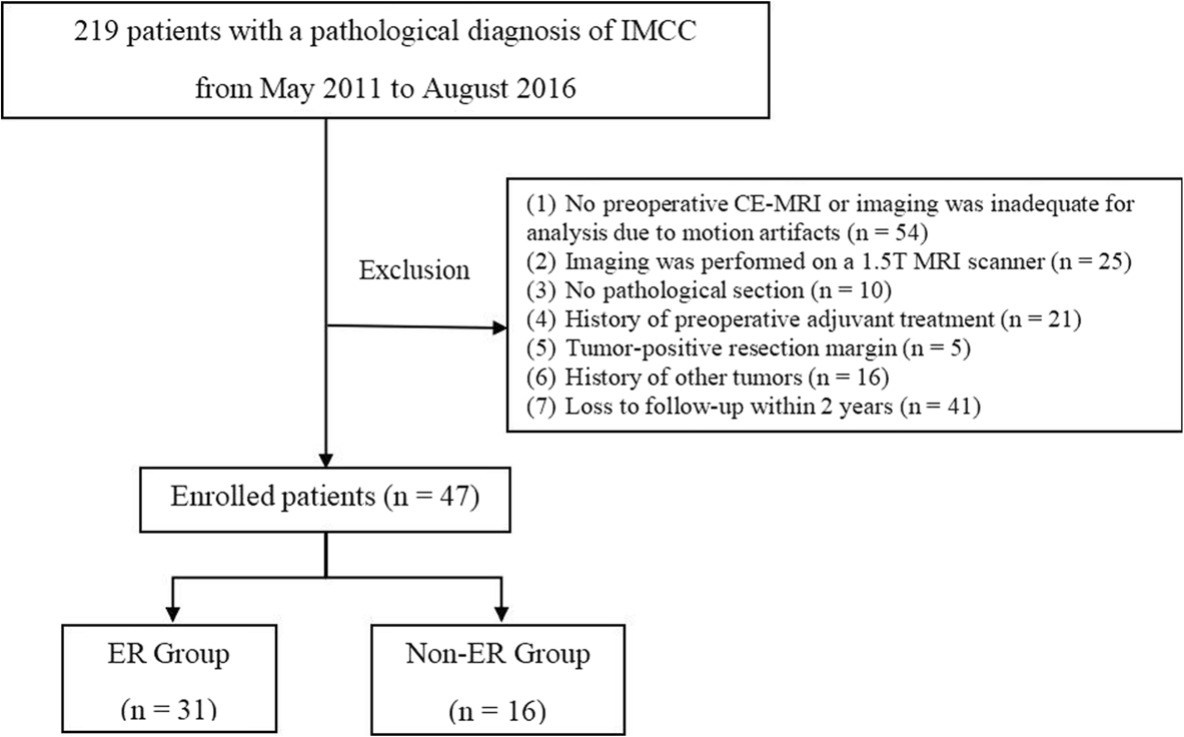
Flowchart of the enrolled patients. IMCC, intrahepatic mass-forming cholangiocarcinoma; MRI, magnetic resonance imaging; CE-MRI, contrast enhanced magnetic resonance imaging; ER, early recurrence

This retrospective study was approved by our Institutional Review Board, and the need for informed consent was waived. We collected electronic medical records from May 2011 to August 2016 at our institution. A total of 219 patients who underwent a curative-intent resection and lymph node dissection, with histopathologically confirmed IMCC, were recruited. Among these patients, the study population was selected using the following inclusion criteria: (a) patients who underwent preoperative liver CE-MRI within 4 weeks of their surgery, (b) patients without a history of previous adjuvant treatment before the surgery, (c) patients with histopathologically proven IMCC and negative resection margin (R0), combined hepatocellular-cholangiocarcinoma were excluded, (d) patients without a history of other tumors, and (e) patients who completed at least 2 years of follow-up. Consequently, 47 patients were enrolled in our study and were divided into an ER group (n = 31) and a non-ER group (n = 16), with ER being defined as the development of intrahepatic or extrahepatic recurrence within 2 postoperative years (Fig. 1).

### Clinical and pathologic characteristics

Clinical and pathological characteristics consisted of age, gender, hepatitis, carcinoembryonic antigen (CEA), carbohydrate antigen 199 (CA199), satellite lesions, maximum tumor diameter (MTD), tumor location, differential degree of tumor, stage and lymph node metastases. Lymph node metastasis was determined by the pathological results of lymph node dissection and preoperative CT/MR imaging. The threshold values chosen for CA199 and CEA levels were based on the normal ranges used at our institution (0-37 U/ml for CA199 and 0-5 ng/ml for CEA).

### Immunohistochemistry

All pathology sections and macroscopic pictures of the resected specimen were retrospectively reviewed. Combining with immunohistochemistry, all 47 patients were confirmed IMCC by post-operative pathology. Immunohistochemical staining was detected on formalin-fixed, paraffin-embedded sections using standard immunohistochemical methods. Then five-micron-thick sections were created, and antibodies specific for EGFR, VEGFR, P53, and Ki67 (Beijing Zhongshan Golden Bridge Biotechnology Co. LTD, China) were used to perform the further immunohistochemical staining. All samples were analyzed by an anatomic pathologist with 10 years of experience, who was unaware of the patient’s outcome. Less than 10% of the positive staining was identified as negative expression, while more than 10% of the positive staining was identified as positive expression.

### Follow-up

All patients underwent contrast enhanced CT or MRI every 3–6 months after surgery in the first 2 years. Images were analyzed to identify ER, which was determined as the presence of new intrahepatic lesions with typical imaging features of IMCC, atypical lesions with histopathological confirmation, or extrahepatic metastasis (lymph node metastases or distant metastasis) within 2 postoperative years.

### Magnetic resonance imaging acquisition and analysis

All patients underwent 3.0 T MRI scans (Signa Excite HDxt, GE Healthcare, Milwaukee, USA) with an eight-element phased-array torso coil. After nonenhanced axial breath-hold T1 weighted imaging, fat suppression T2 weighted imaging (T2WI/FS) and DWI (b-values of 0 and 800 s/mm^2^), contrast enhanced T1-weighted three-dimensional (3D) spoiled gradient echo sequence (LAVA) was performed. Gadodiamide (Omniscan 0.5 mmol/ml; GE Healthcare, Ireland) was injected at a dose of 0.2 mL per kilogram and a rate of 2 mL per second as a bolus by an automatic pump injector and a subsequent 20 mL 0.9% sterile saline flush. Contrast enhanced imaging was performed in the arterial phase (AP) (30 s), portal venous phase (PVP) (60 s), and delayed phase (DP) (180 s). Images of AP, PVP, and DP were all used for radiomics feature extraction and analysis. Additional technical details are provided in Table 1.

**Table 1:** The details of MR imaging sequences parameters

	TR/TE(ms)	FOV (cm)	Slice thickness /space (mm)	Matrix
Breath-hold axial T1WI	250/2.9	34–38	5.0/0.5	288 × 192
Axial T2WI/FS	7000/100	34–38	5.0/0.5	288 × 224
Respiratory-triggered axial DWI	2500/65	34–38	6.0/2.0	128 × 128
CE-MR (multiphase 3D-LAVA sequence)	2.9/1.3	36–42	4.0/0	512 × 512

*Note*: *CE-MR* contrast-enhanced-MR

MR images were reviewed independently by two radiologists who had 5 and 10 years experience in the abdominal MRI, respectively. Both radiologists were blinded to the clinical data of the patients when they evaluated the MR images. They reached a consensus by discussion when there were disagreements. The basic imaging traits potentially associated with ER included lesion shape, contour, biliary dilation, capsular retraction, DWI intensity and the enhancement pattern. The target appearance was peripheral hyperintensity compared to the center on high b-value DWI. The enhancement pattern was assessed using the following subdivisions: (a) gradual enhancement (the enhancement area gradually increased from the periphery to the center of the tumor), (b) persistent enhancement (enhancement remained through all phases), (c) wash in and wash out (hyperenhancement of the AP followed by washout), and (d) minimal or no enhancement.

### Radiomics features extraction and analysis

MR images (AP, PVP, DP, T2WI/FS sequence) were loaded into ITK-SNAP software (version 2.2.0, www.itksnap.org) for 3D manual segmentation. A radiologist with 10 years of MRI experience (reader 1) performed the tumor segmentations in all 47 patients. After 2 weeks, images of all patients were segmented again by reader 1 and another radiologist (reader 2) with 5 years of experience of MRI diagnosis to assess intra−/inter-reader agreement in the feature analysis. All outcomes were based on the features extracted by the first segmentation from reader 1.

Artificial Intelligence Kit software (A.K. software; GE Healthcare, Life Sciences, Beijing, China) was used to extract 396 parameters from each sequence. Those parameters include first order histogram features (*n* = 42), grey-level co-occurrence matrix (GLCM) features (*n* = 144), grey-level run-length matrix features (*n* = 180), Haralick features (*n* = 10), morphological features (*n* = 9) and grey-level zone size matrix features (*n* = 11).

**Fig. 2:**
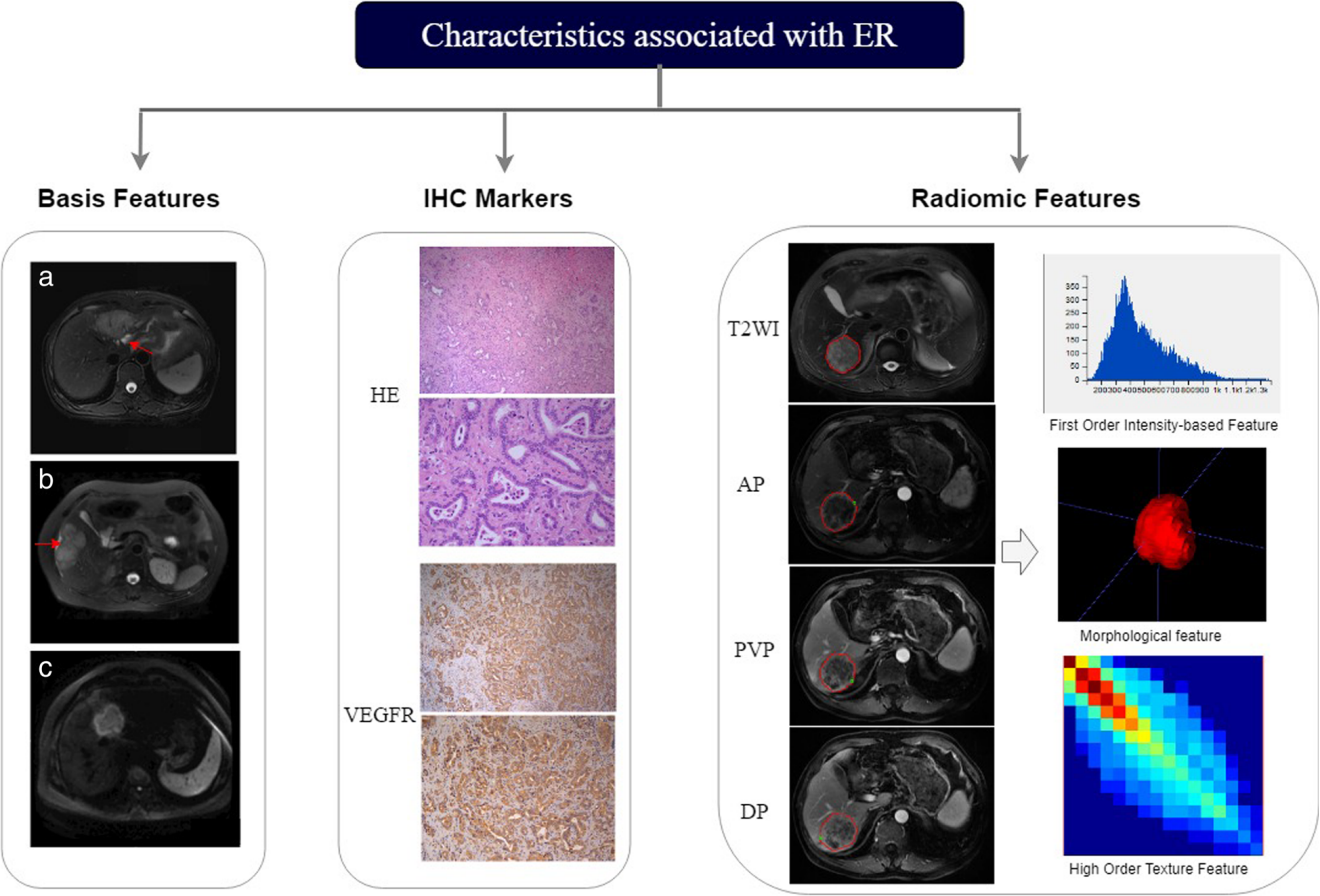
Study workflow. Basic features of biliary dilation on T2WI/FS (**a**), capsular retraction on T2WI/FS (**b**), and target appearance on DWI (**c**). ER, early recurrence; ROI, region of interest; HE, hematoxylin and eosin; VEGFR, vascular endothelial growth factor receptor; T2WI/FS, fat suppression T2-weighted imaging; PVP, portal venous phase; AP, arterial phase; DP, delayed phase; DWI, diffusion weighted image

The proposed parameters were analyzed for consistency and correlation. First, the intraclass correlation coefficient was determined for each parameter for the inter-observer and intra-observer reproducibility test. Features with intraclass correlation coefficient values less than 0.8 were excluded. Second, stratified analyses were conducted using the Wilcoxon signed-rank test to discover the potential association between the remaining parameters and ER status, followed by univariate logistic regression. To keep discriminative parameters, we set a threshold of 0.1. A variance inflation factor was then used to eliminate parameters with high collinearity in a multiple mutual linear situation. Finally, multivariate logistic regression was applied to evaluate the performance of distinguishing ER status in each sequence. Different sequence combinations (AP + PVP, AP + PVP + DP, AP + PVP + DP + T2WI) were also tried to explore the best model using the same methods described above. The validation of each model was performed by using leave-one-out cross-validation. Thus, three predictive models were built: a best performance radiomics model, a clinicoradiologic-pathologic(CRP) model, and a combined model with both selected radiomics features and clinicoradiologic-pathologic features (Fig. 2).

### Statistics

The differences in patient characteristics between the two groups were assessed using t-test or the Mann-Whitney *U* test for continuous variables and the chi-square test or Fisher exact test for categorical variables. Kappa tests were used to determine inter-observer agreement for qualitative MRI features. Kappa values of 0.81–1.00 indicated excellent agreement, 0.61–0.80 signified substantial agreement, and 0.41–0.60 denoted moderate agreement. Receiver operating characteristics (ROC) curves were performed in each model. The area under the curve (AUC) of the ROC curves, accuracy, sensitivity, specificity, positive predictive values (PPVs) and negative predictive values (NPVs) were obtained and comparisons between the performance of final three models were performed using the Delong test. All statistical analyses were performed using R studio Server (Version 3.5.0; RStudio, Inc., Boston, MA, USA) and SPSS (version 20.0; IBM, Armonk, NY, USA). A two-sided *P* value less than 0.05 indicated a statistically significant difference.

## Results

The median age of the patients was 57 years old (range, 35 to 78 years old), and ER was found in 31 (66.0%) patients. The median MTD was 5.73 cm, ranging from 1.5 to 12.8 cm. The median follow-up was 34 months (range, 25 to 87 months). Clinical and pathological characteristics in the ER group and non-ER group are summarized in Table 2. There were no significant differences between the ER group and non-ER group in terms of these characteristics. Radiological features and immunohistochemical markers are listed in Table 3. Among these factors, enhancement patters and VEGFR expression showed significant differences between the ER group and non-ER group (*P* = 0.001 and 0.034, respectively). Additionally, among 33 IMCC patients with gradual enhancement pattern, 26 (78.8%) patients were in the ER group, while all 5 (100%) patients with wash in and wash out enhancement pattern were in non-ER group (*P* = 0.002). The inter-observer agreement for the radiological features showed excellent agreement (k = 0.811–0.849).

**Table 2:** Characteristics of patients

Characteristics	Non-ER (n = 16)	ER (*n* = 31)	P value
Age (years), Mean ± SD	57.69 ± 10.35	56.94 ± 8.83	0.795
Gender, No. (%)			0.763
Male	7 (43.8)	15 (48.4)	
Female	9 (56.2)	16 (51.6)	
Hepatitis, No. (%)			0.074
Present	3 (18.8)	14 (45.2)	
Absent	13 (81.2)	17 (54.8)	
CA199 level (U/ml), No. (%)			0.528
< 37	6 (37.5)	9 (29.0)	
≥ 37	6 (37.5)	18 (58.1)	
Not available	4 (25.0)	4 (12.9)	
CEA level (ng/ml), No. (%)			0.851
< 5	13 (81.3)	23 (74.2)	
≥ 5	2 (12.5)	6 (19.4)	
Not available	1 (6.2)	2 (6.4)	
Satellite lesions, No. (%)			0.316
Absent	15 (93.8)	24 (77.4)	
Present	1 (0.2)	7 (22.6)	
MTD, No. (%)			0.905
< 5	7 (43.8)	13 (41.9)	
≥ 5	9 (56.2)	18 (58.1)	
Lesion location, No. (%)			0.780
Left lobe	9 (56.2)	18 (58.1)	
Right lobe	7 (43.8)	11 (35.5)	
Left and Right lobe	0 (0)	2 (6.4)	
Histologic grade, No. (%)			0.571
Well	1 (6.2)	0 (0)	
Moderate	9 (56.2)	17 (54.8)	
Poor	6 (37.5)	14 (45.2)	
T stage, No. (%)			0.074
I/II	13 (81.2)	17 (54.8)	
III/IV	3 (18.8)	14 (45.2)	
Lymph node metastasis, No. (%)			0.103
Present	1 (6.2)	10 (32.3)	
Absent	15 (93.8)	21 (67.7)	

*Note*: *ER* early recurrence, *CA199* carbohydrate antigen 199, *CEA* carcinoembryonic antigen, *MTD* maximum tumor diameter, *, *P* < 0.05

**Table 3:** Radiological features and immunohistochemistry

	Non-ER (n = 16)	ER (n = 31)	P value
Shape, No. (%)			0.063
Globular	4 (25.0)	1 (3.2)	
Lobulate	3 (18.8)	11 (35.5)	
Irregular	9 (56.2)	19 (61.3)	
Lesion contour, No. (%)			0.770
Well defined	11 (68.8)	24 (77.4)	
Infiltrative	5 (31.2)	7 (22.6)	
Biliary dilation, No. (%)			0.609
Present	7 (43.8)	16 (51.6)	
Absent	9 (56.2)	15 (48.4)	
Capsular retraction, No. (%)			0.465
Present	4 (25.0)	11 (35.5)	
Absent	12 (75.0)	20 (64.5)	
DWI intensity, No. (%)			0.744
Hyperintense	10 (62.5)	17 (54.8)	
Target appearance	4 (25.0)	11 (35.5)	
Slightly hyperintense	2 (12.5)	3 (9.7)	
Enhancement pattern, No. (%)			0.001*
Gradual enhancement	7 (43.8)	26 (83.9)	
Persistent enhancement	3 (18.8)	5 (16.1)	
Wash in and wash out	5 (31.2)	0 (0)	
No or minimal enhancement	1 (6.2)	0 (0)	
EGFR, No. (%)			0.859
Negative	3 (18.8)	8 (25.8)	
Positive	13 (81.2)	23 (74.2)	
P53, No. (%)			0.555
Negative	10 (62.5)	22 (71.0)	
Positive	6 (37.5)	9 (29.0)	
VEGFR, No. (%)			0.034*
Negative	8 (50.0)	5 (16.1)	
Positive	8 (50.0)	26 (83.9)	
Ki67, No. (%)			0.313
Negative	7 (43.8)	9 (29.0)	
Positive	9 (56.2)	22 (71.0)	

*Note*: *ER* early recurrence, *DWI* diffusion weighted image, *EGFR* epidermal growth factor receptor. *VEGFR* vascular endothelial growth factor receptor, *, *P* < 0.05

We began by developing radiomics models based on T2WI/FS, AP, PVP, and DP images separately. The PVP model demonstrated preferable accuracy, sensitivity, specificity, PPV and NPV (0.872, 0.75, 0.936, 0.857, and 0.879, respectively) while it presented a slightly lower AUC (0.841, 95% confidence interval (CI): 0.697–0.984) than that of the AP model (0.871, 95% CI: 0.761–0.981). Next, radiomics models based on multiple sequences were built according to the aforementioned results, including AP + PVP (two sequences with higher AUC), AP + PVP + DP (multi-phase contrast enhanced sequences), and T2WI/FS + AP + PVP + DP (all sequences) models. The AP + PVP + DP model showed superior AUC (0.889, 95% CI: 0.783–0.996) among all the radiomics models, and was used in the follow-up study. This model illustrated that the four most important parameters for predicting ER were AP_skewness and PVP_Variance, both derived from the histogram, as well as AP_ClusterShade_AllDirection_offset7_SD and AP_GLCMEntropy_angle45_offset7 derived from the GLCM. The accuracy, sensitivity, specificity, PPV, NPV and AUC of the seven radiomics models are presented in Table 4.

**Table 4:** Predictive performance of the radiomics model

	Accuracy (95% CI)	Sensitivity	Specificity	PPV	NPV	AUC (95% CI)
AP	0.851 (0.717–0.938)	0.688	0.936	0.846	0.853	0.871 (0.761–0.981)
PVP	0.872 (0.743–0.952)	0.750	0.936	0.857	0.879	0.841 (0.697–0.984)
DP	0.809 (0.667–0.909)	0.750	0.839	0.706	0.867	0.782 (0.619–0.945)
T2WI	0.617 (0.464–0.755)	0.875	0.485	0.467	0.882	0.690 (0.532–0.847)
AP + PVP	0.809 (0.667–0.909)	0.750	0.839	0.706	0.867	0.863 (0.754–0.971)
AP + PVP + DP	0.809 (0.667–0.909)	0.875	0.774	0.667	0.923	0.889 (0.783–0.996)
AP + PVP + DP + T2WI	0.830 (0.692–0.924)	0.688	0.903	0.786	0.849	0.855 (0.737–0.973)

*Note*: *AP* arterial phase, *PVP* portal venous phase, *DP* delay phase, *PPV* positive predictive value, *NPV* negative predictive value, *CI* confidence interval, *T2WI* T2-weighted imaging

**Fig. 3:**
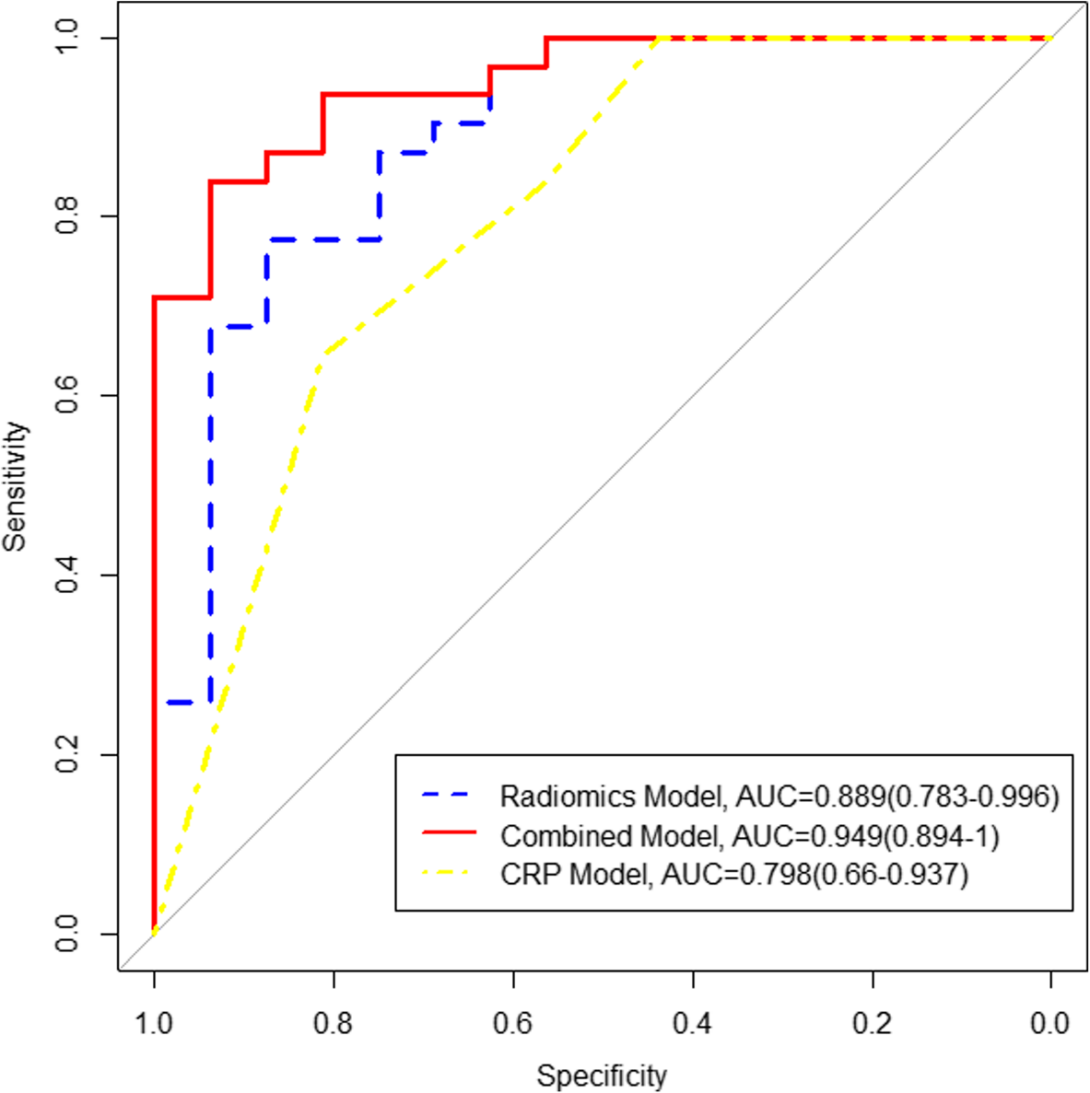
The receiver operating characteristics curves of the radiomics, CRP, and combined models. AUC, area under the curve; CRP, clinicoradiologic-pathological

**Fig. 4:**
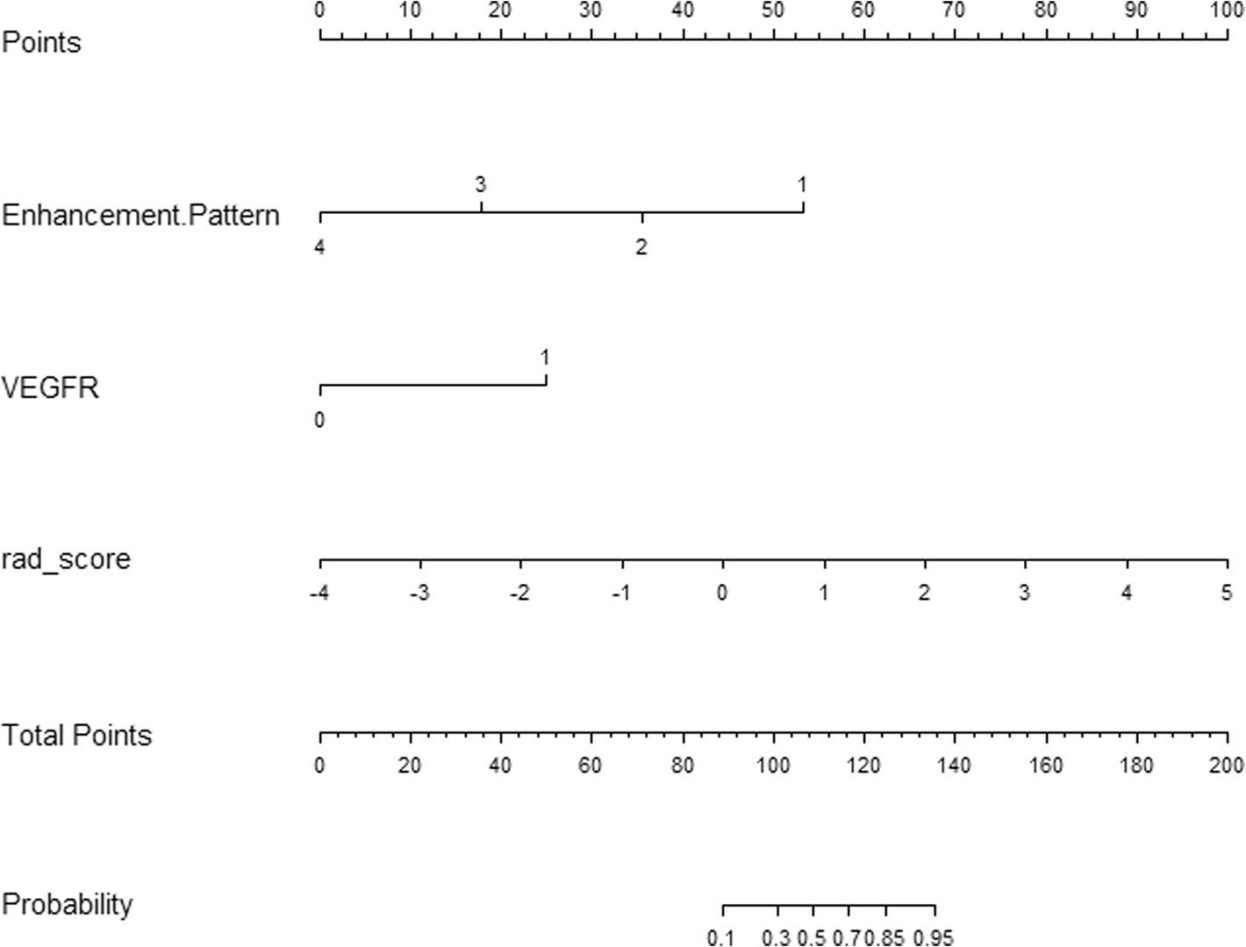
Nomogram with rad_score, enhancement pattern of pre-operative MRI and VEGFR. MRI, magnetic resonance imaging; VEGFR, vascular endothelial growth factor receptor

The clinicoradiologic-pathologic (CRP) model contained enhancement pattern and VEGFR. The combined model incorporated radiomics features, clinicoradiological features and pathological factors. The predictive performance of the CRP model, radiomics model, and combined model are listed in Table 5, and ROCs are shown in Fig. 3. Nomograms for the combined model are presented in Fig. 4. The combined model displayed the best accuracy, sensitivity, specificity, PPV, NPV and AUC (0.872, 0.938, 0.839, 0.750, 0.963 and 0.949, respectively). Also, the combined model significantly improved the predictive performance of the CRP model in predicting ER of IMCC (*P* = 0.009).

**Table 5:** Predictive performance of three models

Model	Accuracy (95%CI)	Sensitivity	Specificity	PPV	NPV	AUC (95%CI)	P value		
							1 vs 2	2 vs 3	1 vs 3
1 Combined model	0.872 (0.743–0.952)	0.938	0.839	0.750	0.963	0.949 (0.894–1.000)	0.247	0.321	0.009*
2 Radiomics model	0.809 (0.667–0.909)	0.875	0.774	0.667	0.923	0.889 (0.783–0.996)			
3 CRP model	0.702 (0.551–0.827)	0.813	0.645	0.542	0.870	0.798 (0.660–0.937)			

Note: The number 1 indicates the combined model, 2 indicates the radiomics model, and 3 indicates the CRP model. Comparisons of the AUC between the three models were made using the Delong test. *CRP* clinicoradiologic-pathologic; combined model, radiomics and CRP model, *AUC* area under the curve, *CI* confidence interval, *PPV* positive predictive value, *NPV* negative predictive value, *, *P* < 0.05

## Discussion

Combining radiomics features, enhancement patterns, and VEGFR led to significant improvements in the AUC, sensitivity, specificity and accuracy for predicting ER compared to the radiomics model or CRP model alone, which indicated that the combination of qualitative and quantitative MRI features along with immunohistochemical markers maximizes the predictive performance of ER. Radiomics models based on CE-MRI sequences (AP, PVP, or DP) showed better specificity and AUC for predicting ER than that of T2WI, though they exhibited relatively lower sensitivity.

Previous studies have investigated ER predictions of IMCC. For instance, Liang et al. developed a novel nomogram to predict the recurrence of ICC with AUC, sensitivity, and specificity values of 0.90, 0.74, 0.89, respectively [26]. This nomogram was achieved based on radiomics and clinical stage, and radiomic signatures extracted only from AP images with a relatively low sensitivity. Jeong et al. established a predictive nomogram of IMCC recurrence based only on clinical characteristics: lymph node metastasis, tumor size, surface antigen of the hepatitis B virus, and Child–Pugh score, with a concordance C index of 0.71 (95% CI: 0.65–0.77) [27]. Our study demonstrated preferable sensitivity and AUC compared with these studies. Furthermore, our combined model that included radiomics features, clinicoradiological and pathological factors was the superior predictive model. This suggested that combining the morphology, quantification of tumor heterogeneity and molecular pathology could better reflect aggressive malignant tumor biology.

There have been an increasing number of studies showing the potential of radiomics based on MR images for diagnosis and prognosis assessment for specific tumors. In our study, the radiomics features were extracted from T2WI/FS, AP, PVP and DP of contrast enhanced images to build the best radiomics model. Radiomics models of AP, PVP and DP all provided better AUC and specificity for predicting ER than that of T2WI/FS, which suggested that CE-MRI contain more potential tumor heterogeneous information. Catharina et al. also revealed the exceptional discriminating ability provided by CE-MRI among T1WI, T2WI, T2WI/short inversion time recovery, and contrast enhanced sequences in differentiating low-grade chondrosarcoma and enchondroma by texture analysis (TA) [28]. Furthermore, the AP + PVP + DP model showed superior AUC among all the TA models, which was consistent with previous studies. Ueno et al. [29] found that a TA model based on texture parameters with several sequences led to a better predictive value than that with a single sequence, demonstrating that multi-phase CE-MRI could provide added value.

We found that VEGFR was a predictor of ER, which was consistent with previous studies. Sang et al. found that ICC with positive VEGFR expression represented aggressive malignancy owing to the mechanism that inhibition of VEGFR-2 expression increased apoptosis and decreased cell proliferation [30]. The enhancement patterns were also related to ER due to the histopathologic basis of fibrous stroma in tumors. Gradual enhancement pattern was relevant to the large amount of fibrous stroma of the tumor and indicated a poor prognosis [31]. Small IMCC with diameter less than 3 cm in the cirrhotic liver showed atypical wash in and wash out enhancement pattern frequently [32]. IMCC with wash in and wash out enhancement pattern demonstrated less central fibrous stroma and more cellular areas than that with gradual enhancement pattern; also, hyperenhancement on AP was an independent factor for longer survival.

Whether MTD and CA199 levels could serve as a predictor of ER is currently controversial, perhaps due to the heterogenous population of IMCC patients. Several studies reported that no associations were found between MTD and IMCC prognosis [33]. Nevertheless, other studies found that MTD was associated with ER [27, 34]. Similarly, a few previous studies found CA199 to be a preoperative predictor of prognosis [34, 35], while others removed CA199 as an independent prognostic factor of ER [27]. In our study, MTD and CA199 levels displayed no significant correlation with ER.

Our study had some limitations. First, since it was a retrospective study and was performed in a single center, thus lacking the heterogeneity of MR images and the cohort population of other institutions, selection bias may exist. We will explore the prediction model using MR images from multiple centers in the future. Other limitations of this study were the relatively small sample size cohort due to the incidence of IMCC and the need to obtain pathological sections for immunohistochemistry. Finally, we only developed predictive models for ER without including long-term survival analysis. Prediction of long-term survival should be included in future studies.

## Conclusions

Our study show that the combined model was the superior predictive model of ER compared with radiomics or CRP model alone. Combining qualitative and quantitative MRI features and VEGFR might be useful for predicting ER and guide personalized treatment in patients with IMCC.

## Data Availability

The datasets used and/or analyzed during the current study are available from the corresponding author on reasonable request.

## Abbreviations

3D: Three-dimensional
AP: Arterial phase
CA199: Carbohydrate antigen 199
CEA: Carcinoembryonic antigen
CE-MRI: Contrast enhanced MRI
CI: Confidence interval
CRP: Clinicoradiologic-pathological
DP: Delayed phase
EGFR: Epidermal growth factor receptor
ER: Early recurrence
GLCM: Grey-level co-occurrence matrix
ICC: Intrahepatic cholangiocarcinoma
IMCC: Intrahepatic mass-forming cholangiocarcinoma
MTD: Maximum tumor diameter
PVP: Portal venous phase
ROI: Region of interest
T2WI: T2-Weighted imaging
TA: Texture analysis
VEGFR: Vascular endothelial growth factor receptor
